# Medical education too: sexual harassment within the educational context of medicine – insights of undergraduates

**DOI:** 10.1186/s12909-021-02497-y

**Published:** 2021-02-01

**Authors:** Eva Schoenefeld, Bernhard Marschall, Berit Paul, Helmut Ahrens, Janina Sensmeier, Jan Coles, Bettina Pfleiderer

**Affiliations:** 1 Institute of Medical Education and Students’ Affairs, Medical Faculty, Westfalian Wilhelm University of Münster, Malmedyweg 17-19, 48149 Münster, Germany; 2 Münster medical students, Westfalian Wilhelm University of Münster, Münster, Germany; 3 Women’s Health MMed Women’s Health, MBBS DCH GCHPE, Monash Centre for Scholarship in Health Education, Faculty of Medicine, Nursing and Health Sciences, Monash University, Clayton, Victoria Australia; 4 Institute for Clinical Radiology, Medical Faculty, Westfalian Wilhelm University of Münster, Medical Women’s International Association, Münster, Germany

**Keywords:** Sexual harassment, Gender inequalities, Discrimination, Undergraduate education

## Abstract

**Background:**

Assessment of the presence and characteristics of sexual harassment in academic medicine is a global issue. Only limited international data are available so far.

**Methods:**

Aim: To assess the extent of sexual harassment and identify the perpetrators in the student population of the medical school of Münster, Germany.

A survey was undertaken, using the Medical Women’s International Association sexual harassment questionnaire translated into German. The anonymous online questionnaire was sent as a link to all medical undergraduates at Münster Medical School via a mailing list between 1 October and 30 November 2018. Identifying or potentially identifying data were not collected.

Data were analysed by descriptive statistical methods such as categorical variables. Baseline characteristics, e.g. answers by male or female medical students, were correlated with their individual sexual harassment experiences and perpetrator groups by means of univariate analysis.

**Results:**

A total of 2162 medical students were asked to participate, with 623 (28.8%) completing the survey. Sexual harassment is a significant issue among medical students at Münster Medical School with over half (58.9%) of all undergraduates being exposed to sexually harassing behaviour. In total, 31.8% of all participants reported having experienced unwanted physical sexual contact such as unwanted physical touching, with 87.6% of the victims being female. Overall, 41.3% personally experienced verbal sexual harassment of which 87.4% were female. Furthermore, 8.5% of undergraduates faced forced sexual contact such as oral, anal or vaginal penetration, intercourse and rape, with all victims being female. Perpetrators in these cases were mostly male medical superiors (7.0%) and male patients (18.3%). In general, most perpetrators were patients, followed by medical superiors and educators, and less frequently by colleagues.

**Conclusions:**

Sexual harassment in medical education and the medical workplace is a significant problem in a German medical school. Most students experiencing sexual harassment are females. Female students also experience the more serious forms of sexual harassment more often.

**Supplementary Information:**

The online version contains supplementary material available at 10.1186/s12909-021-02497-y.

## Background

This study investigates the prevalence of sexual harassment among undergraduate medical students in a German medical school. The World Health Organization (WHO) definition of sexual harassment was used, namely that ‘Sexual harassment means any unwelcome sexual advance, request for sexual favours, or other verbal or physical conduct of a sexual nature, when it interferes with work, is made a condition of employment, or creates an intimidating, hostile or offensive work environment’ [1].

Sexual harassment results in physical and psychological suffering [1–6] such as depression, social isolation, fear and associated cardiovascular symptoms. Four out of ten female physicians from the UK reported similar findings [7, 8] and physicians at the Charité in Berlin (Germany) further corroborated this [9]. Moreover, when sexual harassment occurred, it was often not reported [10, 11].

The goal of our study was to determine the occurence sexual harassment in undergraduates at the medical school in Münster, Germany, where 60–70% of medical students are female. Münster Medical School is one of the largest in Germany. Confronting individual stories of sexual harassment in Münster Medical School was the driver for this study. A questionnaire on this topic was conducted among medical undergraduates to better describe the problem with baseline data to assist and inform future educational practice and policy.

## Methods

A validated sexual harassment questionnaire in the medical working environment is not available in German speaking countries. We translated an international questionnaire from the Medical Women’s Medical Association (MWIA) into German (http://www.europarl.europa.eu/RegData/etudes/STUD/2018/604949/IPOL_STU(2018)604949_EN.pdf). The MWIA drafted the questionnaire for their own global survey in 2017. The original English version of the survey can be found in the supplementary material. The MWIA study and questionnaire used was approved by the Monash University Human Research Ethics Committee, Melbourne, Australia (Project ID 10064) and was designed by an international team of experts in medical education led by Prof. Jan Coles. The German version of the questionnaire was anonymous and did not collect any identifying or potentially identifying personal data. For this reason, Human Research Ethics Committee approval was not required after discussion with the local ethics panel.

The German questionnaire was distributed via a mailing list of all our medical students as a link. We started, after asking for gender affiliation, with definitions and legal aspects concerning sexual harassment and bullying as well as equality under public law in Germany. The definition of sexual harassment was in accordance with the WHO definition outlined in the section headed “background” (https://www.telegraph.co.uk/news/2018/11/01/nhs-needs-metoo-moment-stamp-sexual-harassment-doctors-union/) [12–14]. We also applied definitions from the German Penal code. The Penal Code in Germany starts with an anti-discrimination statement [15]:

1. Prohibition of Discrimination Under Civil Law
2. Any discrimination on the grounds of race or ethnic origin, sex, religion, disability, age or sexual orientation shall be illegal when founding, executing or terminating civil-law obligations [14], and further refers to employer and employee duties and rights.
3. Penal Law: Enforcement
4. Where a breach of the prohibition of discrimination occurs, the disadvantaged person may, regardless of further claims being asserted, demand that the discriminatory conduct be stopped. Where other discrimination is to be feared, he or she may sue for an injunction.
5. Where a violation of the prohibition of discrimination occurs, the person responsible for committing the discrimination shall be obligated to compensate for any damage arising therefrom. This shall not apply where the person committing the discrimination is not responsible for the breach of duty. The person suffering discrimination may demand appropriate compensation in money for the damage, however not for economic loss.
6. Claims in tort shall remain unaffected.
7. The person responsible for committing the discrimination shall not be permitted to refer to an agreement which derogates from the prohibition of discrimination.
8. Any claims arising from Subsections (1) and (2) must be asserted within a period of 2 months. After the expiry of the time limit the claim may only be asserted when the disadvantaged person was prevented from meeting the deadline through no fault of their own.”

A total of 2162 medical students, 1427 females, at the Westfalian University of Münster were asked to participate deliberately and anonymously in the online survey between 10 October and 30 November 2018.

The survey was divided into two sections: one contained ten statements on general and individual experiences and different forms of sexual harassment, including verbal and sexual contact, and forced physical sexual contact. The second section detailed the frequencies of specific sexual harassment experiences and perpetrator groups. Each part finished with a free text option on personal narratives, consequences and ideas for intervention. Inclusion criterion was completed data set. Incompletely answered surveys were excluded. Demographic data solely concerned gender affiliation. Agreement or disagreement with ten different statements followed in Part 1 of the questionnaire. Answers could be given with a five step Likert scale.

Part 1: Statements to (dis-)agree with included: ‘Did you observe sexual harassment against males/females within your medical educational field?’ Response options ranged from: I agree completely; I agree partially; I do not know; I disagree partially; and I disagree completely.

Statements in Part 2 contained: ‘Have you ever felt that your job or your future job was dependent on you performing an unwanted sexual behaviour?’ They could choose between ‘never’ to ‘once’, ‘two to five times’ and ‘more than five times’.

In Part 2, questions concerning people who were responsible for sexual harassing actions including ‘manager or supervisors?’, ‘colleagues?’, ‘patients?’ or ‘other group of people’ were asked.

A contingency plan was put in place to help survey respondents deal with the potential for emotional and/or psychological distress resulting from completing the survey and reliving potentially traumatic experiences. The Medical Faculty in Münster has a helpdesk for students and the leading psychologist, Mrs. Janina Sensmeier, is a co-author of this manuscript. In addition, a special support consultation was available in case of need as part of the study.

### Statistical analysis

Continuous data are presented as the mean ± standard deviation (range) and categorical data are presented as the frequency (percentage). Continuous data were analysed using the Mann–Whitney U test. Paired continuous data were compared using the Wilcoxon signed rank test. Proportions were compared using the Chi-square or Fisher-exact test, as appropriate and as necessary. Examples of qualitative data are presented but the formal analysis of the free text is yet to be completed. Data of partial and complete agreement were collapsed, as were partial and complete disagreements.

## Results

### Victims

Six hundred twenty-three (28.8%) of the 2162 medical students answered the online questionnaire completely. Four hundred sixty-seven (74.8%) of them were female, 156 (25.1%) were male. None defined themselves as ‘diverse’. In Germany, the category of gender affiliation ‘diverse’ means lesbian-gay-, bi-sexual and transgender or queer individuals (LGBTQs). Eighty-nine surveys were excluded for being incomplete; 50 of them (56.2%) were from female participants.

Nearly a quarter (24.6%) observed different forms of sexual harassment (Table 1). The same proportion (24.6%) was aware that sexual harassment is present in medicine in general. When asked about personal experiences of sexual harassment, the percentage increased to 58.9% of the 623 responding. The key results are summarised in Table 2.

**Table 1:** Various forms of sexual harassment reported from undergraduates stratified by sex of our Münster medical school

Statements	Answers of (partial) agreement (n)	Females (partial) agreement (n%)		Males (partial) agreement (n%)	
Observed sexual harassment in the medical field in general	365	273	74.8	92	25.2
Observed sexual harassment at the educational setting^a^	153	114	74.5	39	25.5
Personally experienced sexual harassment	154	135	87.6	19	12.3
Job and career advancement was coupled with behaving in the desired way e.g. wearing clothes with deep neckline	74	70	94.6	4	5.4
Physical sexual harassment, e.g. unwanted touching	198	193	97.5	5	2.5
Verbal sexual harassment, e.g. sexualized comments, jokes	257	192	74.7	65	25.3
Comments on sexual orientation	46	35	76.1	11	23.9
Unnecessary intimate examinations	60	55	91.6	5	8.4
Forced sexual contact	53	53	100	0	0
Forced sexual intercourse	8	8	100	0	0

^a^Educational setting encompasses the non-clinical setting in seminars and simulation-based assessments and trainings, as well bed-side teaching at Muenster medical school

Explanation: Data of disagreement and “I do not know” are not shown

**Table 2:** Total percentage and percentage of different forms of experienced sexual harassment and statement results stratified by sex

Key Results	Total^a^	Female^b^	Male^b^
Reported personally experienced verbal sexual harassment	41.3	87.4	12.6
Reported personally experienced physical sexual harassment	31.8	87.6	12.4
Reported personally experienced forced sexual contact/intercourse	8.5	94.0	6.0
Statement 1: Our undergraduates are aware of sexual harassment within the medical field.	74.8	74.6	25.4
Statement 2: Our female students have personally experienced sexual harassment in general.	21.7	87.6	12.3
Statement 3: Physical sexual harassment and forced sexual contact is experienced by our female students.	31.0	97.5	2.5
Perpetrator groups as mentioned by students in the survey and sex of victims of sexual harassment stratified by			
Patients	18.3	88.4	11.6
Superiors	7.0	93.7	6.3

^a^Related to the total number of respondents (*n* = 623)

^b^Related to the total number of those who have personally experienced either verbal, physical or forced sexual contact

The first three items present prevalence of personally experienced forms of harassment, while the statements combined several responses to one item

Of note, those who experienced physical sexual harassment and/or forced sexual contact were all females. In the free text answers, further descriptions of physical sexual harassment such as ‘unwanted touch’ were found. Physical assaults were ‘unambiguous’ for the female undergraduates, but they reported being unable to say ‘no’ or ‘stop’.

### Perpetrators

Over 41% (41.3%) of our students experienced inappropriate sexual comments on their appearance, clothing, sexual orientation or behaviour. Less frequently, offending verbal assaults occurred electronically via email (1%) or short messaging (3.7%). Only 2.4% experienced offending phone calls. In 19.3% of cases, patients were involved, in nearly 10 % educators/superiors (9.8%) or colleagues (9.3%) were involved (Table 3).

**Table 3:** Distribution and percentages of perpetrators of different forms of sexual harassment

Forms of sexual harassment	Group of responsible perpetrators	Answers from female medical students^a^ n (%)	Answers from male medical students^a^ n (%)	Overall answers “yes”^b^ n (%)
Verbal sexual harassment	Superiors, educators	58 (95.1)	3 (4.9)	61 (9.8)
	Patients	110 (91.7)	10 (8.3)	120 (19.3)
	Colleagues	44 (75.9)	14 (24.1)	58 (9.3)
Physical sexual harassment	Superiors, educators	24 (92.3)	2 (7.7)	26 (4.2)
	Patients	92 (85.2)	16 (14.8)	108 (17.3)
	Colleagues	29 (85.3)	5 (14.7)	34 (5.5)
Forced sexual contact	Superiors, educators	15 (93.8)	1 (6.2)	16 (2.6)
	Patients	15 (88.2)	2 (11.8)	17 (2.7)
	Colleagues	5 (100)	0 (0)	5 (0.8)

^a^Related to the total number of those who had experienced a particular form of sexual harassment

^b^Related to the total number of respondents (*n* = 623)

Explanation: Data “no assault or contact” and “I do not know” are not shown

Analysis of the free texts showed that, in the case of patients acting as perpetrators, the majority were ‘over 50-year-old males’ asking for repetitive intimate examination or exhibitionism (7.5%). However, victims were not always sure this behaviour was inappropriate: ‘maybe he touched my breast accidentally’. Free texts revealed misbehaviour of a male surgeon and educator towards his female student trainees several times. He came extremely close, so that the female trainees and students were caught between him and the OR table.

Legally punishable acts according to the German Penal Code [15]were reported in 9.8% of cases in this survey with 8.5% of the students willing to describe the assaults. Enforced sexual intercourse was reported in eight questionnaires (1.3% of the collective) without free text input. The perpetrators which were reported were, in three cases, medical superiors (37.5%) and educators (37.5%), respectively and in one case, a male patient (12.5%). ‘Other’ perpetrators were not further specified in the free text section.

## Discussion

This survey shows that sexual harassment in medical education and the medical environment exists as an important problem among undergraduate education at a large German medical school in Münster. Medical education contains clinical workplace-based teaching and assessment and simulation, skills training, and summative and formative assessment settings where sexual harassment may take place. Those who reported having personally experienced some form of sexual harassment (58.9%) were mostly female (87.6%), while perpetrators are mostly males (89.7%) and also included patients (18.3% for sexually harassing behaviour). We observed an increase when items and questions were repeated or triggered the individual perspective on that item. This is in accordance with findings of a large national survey on violence against women in Germany in 2004 [14].

Our results in undergraduates corroborate similar findings concerning physicians from a German hospital in Berlin, other European countries and the US [9, 11] (https://www.telegraph.co.uk/news/2018/11/01/nhs-needs-metoo-moment-stamp-sexual-harassment-doctors-union/). The issue of sexual harassment in medicine is a long-standing problem, and the potential negative impact on a patient’s treatment and physician’s well-being is recognised [12, 13]. American psychiatrists reported that women who experienced sexual harassment struggle in isolation in their working environment and were not able to achieve their potential in their career and research fields [10]. In those women with trauma symptoms, only 1–7% had filed a formal complaint; possibly due to a lack of role models or anticipated lack of success. The symptoms caused by the sexual harassment were stress, depression, obesity, chronic illness, an increased absence from the workplace and even cardiovascular diseases. Fifty per cent were bullied by a colleague, 30% by a patient. Fort per cent experienced sexual harassment by superiors in Ireland. Our results are further supported by a position paper of the Irish Medical Organisation (IMO) (https://www.telegraph.co.uk/news/2018/11/01/nhs-needs-metoo-moment-stamp-sexual-harassment-doctors-union/). They also asked for experiences and differentiated female and male responses: 26.2% suffered from gender-based harassment in the last 2 years (31.3% were females and 15.4% were males). Sexual harassment occurred in 18.3% within the last 2 years. They found that discrimination and sexual harassment influenced specialty choice. Surgery was the only specialty in this study where the respondents felt that gender had career implications. Concerning the perpetrator groups, Irish doctors showed a different impact. Fifty per cent of the females were bullied by colleagues; 33% by patients and less by superiors.

Our data also indicate that the more severe forms of sexual harassment such as unwanted physical contact and forced physical contact have patients as the main perpetrator, while sexual assault has superiors and educators as the main perpetrators. This suggests that different strategies for education may be needed targeting patients, staff and supervisors and not limited to students.

To the best of our knowledge no other study concerning undergraduates has been published so far in Germany [11, 14]. Our data clearly demonstrate that the problem exists from early on; starting at undergraduate level and is not confined to those working as physicians. This problem exists not only in Germany but globally and warrants a raised awareness, reaction and reflection on this challenging issue to better support and educate undergraduates. It may be assumed that, following sexual harassment, undergraduates will suffer similar symptoms as reported by physicians [2, 4, 6]. Moreover, we believe that the incidence of sexual harassment is underreported and that measures must be put in place to interrupt the vicious cycle of silence. Interventions, policies and recommendations must be put in place to instil sustainable change.

This study highlights that sexual harassment is an issue at our faculty, but the main limitation of this trial may be some pressure of time in achieving awareness. We omitted conducting a pilot test of the questionnaire; especially concerning its translation. Another limitation that may cause bias is the relatively low response rate of 28.8%.

At Münster Medical School, as a consequence of our findings, an independent voluntary task force of important stakeholders at the medical faculty was formed. Main and consensus goals of the task force were based on the outcomes of this survey among undergraduates and in accordance with the three key recommendations of the Irish position paper [11]. After the identification of the extent, a first meeting with managers of all levels had already been conducted and next measures discussed. We plan training related to sexual harassment on all levels, including physicians as well as students and nursing staff. Communication and reflection on sexual harassment will increase an awareness campaign as started by our task force. Awareness of sexual harassment and its prevention will be emphasised as a part of the development of professional behaviours among our medical students. We are their role models as medical educators and supervisors, and we must work towards eliminating sexual harassment amongst our students by awareness, reflection and communication.

## Conclusion

Sexual harassment is an issue within our local medical educational settings due to a lack of awareness, reflection and communication. It is an interprofessional challenge, and female students experience most assaults. Our task is to take care and create transparency and eliminate sexual harassment.

## Supplementary Information

**Additional file 1.** MWIA Sexual Harassment Survey. IfAS Fragebogen zu Sexismus und sexueller Belästigung.

## Data Availability

The datasets generated and/or analysed during the current study are not publicly available due to anonymous request of the survey and deliberate participation but are available from the corresponding author on reasonable request.
