# Supplementary material for: Medical education too: sexual harassment within the educational context of medicine – insights of undergraduates
Source: BMC Med Educ. 2021 Feb 1;21:81. doi: 10.1186/s12909-021-02497-y (PMC7852293; doi:10.1186/s12909-021-02497-y)
Supplement: Supplementary file 1 — Additional file 1. MWIA Sexual Harassment Survey. IfAS Fragebogen zu Sexismus und sexueller Belästigung. [file 12909_2021_2497_MOESM1_ESM.zip › 2020_07MWIA_Sexual_Harassment_SurveyR5.pdf]

MWIA Sexual Harassment Survey

Your MWIA Region is?

❍ Northern Europe
❍ Central Europe
❍ Southern Europe
❍ North America
❍ Latin America
❍ Near East and Africa
❍ Central Asia
❍ Western Pacific

What country are you working or studying in?

Please state your age in years

You are?

❍ A medical student
❍ A doctor still in training (e.g. intern, registrar, house medical officer)
❍ A doctor who has completed their training

How many years have you worked as a doctor?

❍ 0
❍ 1-5
❍ 6-10
❍ 11-15
❍ 16-20
❍ more than 20

Where do you do your medical work? Please check all that apply.

❑ In a government hospital or clinic
❑ In a private hospital or clinic
❑ In a Non Government Organisation run hospital or clinic
❑ In a University or other education organisation
❑ Other, please state ____________________

According to the country you work in, is your current medical work or medical student placement in a?

❍ Rural location
❍ Urban location

The next section asks questions on your experience of sexual harassment in your workplace. If you are a medical student, this means any site related to your medical studies. If you are a doctor, this means any site where you work as a doctor.

First we need to say what we mean by sexual harassment. The World Health Organization defines sexual harassment in following way: Sexual harassment means any unwelcome sexual advance, request for sexual favours, or other verbal or physical conduct of a sexual

nature, when it interferes with work, is made a condition of employment, or creates an intimidating, hostile or offensive work environment. Most cases of sexual harassment fall into one of two categories:

- Hostile Work Environment: verbal or other conduct of a sexual nature that interferes with work, or creates an intimidating, hostile or offensive work environment.
- Quid Pro Quo: acceptance or rejection of sexual advances, requests for sexual favours, or other verbal or physical conduct of a sexual nature that is used as a basis for an employment decision, or is made a condition of continued employment.

  http://www.un.org/womenwatch/osagi/pdf/who.pdf

Sexual harassment at your study/work place

	Your response to the statement				
	strongly disagree	disagree	neither agree or disagree	agree	strongly agree
Sexual harassment occurs in the medical workplace	❍	❍	❍	❍	❍
Sexual harassment occurs at my place of work	❍	❍	❍	❍	❍
I have personally witnessed sexual harassment female medical students in my workplace	❍	❍	❍	❍	❍
I have personally witnessed sexual harassment of male medical students in my workplace	❍	❍	❍	❍	❍
I have personally experienced sexual harassment of female doctors in my workplace	❍	❍	❍	❍	❍
I have personally witnessed the sexual harassment of male doctors in my workplace	❍	❍	❍	❍	❍
I have personally experienced sexual	❍	❍	❍	❍	❍

harassment in my workplace					
I have experienced work-related sexual harassment by phone	❍	❍	❍	❍	❍
I have experienced work-related sexual harassment via social media	❍	❍	❍	❍	❍
I have experienced work-related sexual harassment online	❍	❍	❍	❍	❍

Sexual harassment frequency and persons responsible

	Frequency				Person most often responsible for harassment (pick one if you answered once or more)				
	never	once	2-5 times	>5 times	Manager or supervisor	Clinical peer or colleague	Patient	Patient family member or friend	Other
Have you ever felt that your job or your future job was dependent on you performing an unwanted sexual behaviour?	❍	❍	❍	❍	❍	❍	❍	❍	❍
Have you experienced someone coming unnecessarily physically close to you in a sexual way?	❍	❍	❍	❍	❍	❍	❍	❍	❍
Have you ever experienced question or comments about your appearance that were in an unwanted or sexual way?	❍	❍	❍	❍	❍	❍	❍	❍	❍
Have you ever experienced sexually offensive phone calls at work or related to work?	❍	❍	❍	❍	❍	❍	❍	❍	❍
Have you ever experienced sexually	❍	❍	❍	❍	❍	❍	❍	❍	❍

offensive text messages at work or related to work?									
Have you ever experienced unwanted sexually offensive emails at work or related to work?	❍	❍	❍	❍	❍	❍	❍	❍	❍
Have you ever experienced offensive remarks about your sexual orientation?	❍	❍	❍	❍	❍	❍	❍	❍	❍
Have you ever been asked to perform unnecessary or repeated physical examination of a sexual area (e.g. genital area)?	❍	❍	❍	❍	❍	❍	❍	❍	❍
Has someone coerced or forced you to have physical contact that was unwanted and sexual (e.g. touching, kissing)?	❍	❍	❍	❍	❍	❍	❍	❍	❍
Has someone coerced you to have	❍	❍	❍	❍	❍	❍	❍	❍	❍

sexual intercourse that was unwanted (e.g. attempted or successful oral, anal or vaginal intercourse) ?									

Gender of person/s responsible for your experience/s of sexual harassment?
- ❍ Male
- ❍ Female
- ❍ Both male and female
- ❍ Does not apply

Are there any comments you would like to make to help us to better understand sexual harassment in your workplace?

What policy is available to prevent female medical students and doctors from experiencing sexual harassment? Please tick all that apply
- ❍ None
- ❍ My workplace has policy
- ❍ My university has policy
- ❍ My training organization/college has policy
- ❍ The government of the country where I work has policy
- ❍ International organizations such as MWIA, WHO, IFMSA have policy

Does policy act as a deterrent of sexual harassment in your experience?
- ❍ Yes
- ❍ Maybe
- ❍ No

The Medical Women's International Association has the following Resolutions on sexual harassment: Resolution No. 5. Violence against Health workers. 2016 (Proposed by Nigeria) Whereas patient-initiated violence against health workers has serious health effects on the health workers and the community MWIA resolves that health workers are entitled to work free from occupational stress and threat (in a safe environment, free from harassment, discrimination, violence, verbal and physical bullying).

Would you like to propose any further resolutions on sexual harassment? Please enter your suggestion in the text box.

How could MWIA work to prevent the sexual harassment of women medical students and doctors?

What initiatives could MWIA undertake to help prevent and improve responses to sexual harassment in the medical workplace?

Any other comments you would like to make?

Are you interested in participating in a follow-up Skype interview about your experiences of sexual harassment in the workplace? Please enter your email below so that the research team can contact you.
