# Supplementary material for: Medical education too: sexual harassment within the educational context of medicine – insights of undergraduates
Source: BMC Med Educ. 2021 Feb 1;21:81. doi: 10.1186/s12909-021-02497-y (PMC7852293; doi:10.1186/s12909-021-02497-y)
Supplement: Supplementary file 1 — Additional file 1. MWIA Sexual Harassment Survey. IfAS Fragebogen zu Sexismus und sexueller Belästigung. [file 12909_2021_2497_MOESM1_ESM.zip › Fragebogen_Gender_IfASR5.docx]

Liebe Studierende,

wir möchten mit diesem Fragebogen eine Erhebung zu einem wichtigen Thema starten: Es geht um sexuelle Belästigung und Sexismus.

Und zu allererst glauben wir, dass Sie und uns dieses Thema etwas angeht!

Wenn darüber gesprochen und diskutiert werden soll, gehört zur Voraussetzung, sich zunächst klar werden, über welchen Umfang von Sexismus wir sprechen. Das ist der „Auslöser“ für diesen Fragebogen.

Deshalb: Beantworten Sie ihn bitte und motivieren Sie möglichst viele Ihrer Kommilitonen und Kommilitoninnen!

Ohne oder mit geringem Rücklauf fehlt uns die Grundlage einer Auseinandersetzung mit dem Thema!

Vielen Dank!

Der Fragebogen ist eine Übersetzung aus dem Englischen und wird in Australien allgemein im medizinischen Kontext verwendet. Mit herzlichem Dank an Frau Professorin Bettina Pfleiderer für die Zurverfügungstellung!

Auf den folgenden Seiten wird nach Ihrer Erfahrung mit sexueller Belästigung an Ihrem Studien-, Ausbildungs- und Arbeitsplatz gefragt.

Für Sie als Medizinstudierende/r sind alle Ihre Ausbildungsorte gemeint (nicht nur am UKM).

Doch an erster Stelle gilt es die Definition von Sexismus und von sexueller Belästigung klarzustellen:

Sexismus ist…jede Art der Diskriminierung, Unterdrückung, Verachtung und Benachteiligung von Menschen aufgrund ihres Geschlechts sowie für die Ideologie, die dem zugrunde liegt. Sexismus finde sich in psychischen Dispositionen, in Vorurteilen und Weltanschauungen ebenso wie in sozialen, rechtlichen und wirtschaftlichen Regelungen, schließlich auch in der Form faktischer Gewalttätigkeit und Ausschließung im Verhältnis der Geschlechter sowie auch in der Rechtfertigung dieser Gewaltakte und -strukturen durch den Verweis auf eine ‚naturgegebene‘ Geschlechterdifferenz. (Quelle: *Brockhaus-Enzyklopädie, 30 Bde., Gütersloh 200621, Bd. 25, S. 106.)*

Sexuelle Belästigung am Arbeitsplatz ist eine Benachteiligung im Sinne des [Allgemeinen Gleichstellungsgesetzes](https://de.wikipedia.org/wiki/Allgemeines_Gleichstellungsgesetz). In § 3 wird sie definiert als „…ein unerwünschtes, sexuell bestimmtes Verhalten, wozu auch unerwünschte sexuelle Handlungen und Aufforderungen zu diesen, sexuell bestimmte körperliche Berührungen, Bemerkungen sexuellen Inhalts sowie unerwünschtes Zeigen und sichtbares Anbringen von pornographischen Darstellungen gehören, bezweckt oder bewirkt, dass die Würde der betreffenden Person verletzt wird, insbesondere wenn ein von Einschüchterungen, Anfeindungen, Erniedrigungen, Entwürdigungen oder Beleidigungen gekennzeichnetes Umfeld geschaffen wird.“

**Sexuelle Belästigung an Ihrem Studien-/Arbeitsplatz**

| Statement | Ich stimme vollständig zu. | Ich stimme überwiegend zu. | Ich stimme weder zu noch lehne ich es ab/Ich habe keine festgefügte Meinung. | Ich lehne das überwiegend ab. | Ich lehne das vollständig ab. |
| --- | --- | --- | --- | --- | --- |
|  | + + | + | +/- | - | - - |
| 1. Sexuelle Belästigung kommt im medizinischen Arbeitsumfeld vor. |  |  |  |  |  |
| 2. Sexuelle Belästigung kommt an meinem Arbeitsplatz vor. |  |  |  |  |  |
| 3. Ich habe persönlich sexuelle Belästigung gegenüber weiblichen Medizinstudierenden beobachtet. |  |  |  |  |  |
| 4. Ich habe persönlich sexuelle Belästigung gegenüber männlichen Medizinstudierenden beobachtet. |  |  |  |  |  |
| 5. Ich habe persönlich sexuelle Belästigung von Medizinerinnen beobachtet. |  |  |  |  |  |
| 6. Ich habe persönlich sexuelle Belästigung von Medizinern beobachtet. |  |  |  |  |  |
| 7. Ich habe persönlich sexuelle Belästigung an meinem Arbeitsplatz erfahren. |  |  |  |  |  |
| 8. Ich habe sexuelle Belästigung am Telefon im Arbeitskontext erfahren. |  |  |  |  |  |
| 9. Ich habe sexuelle Belästigung über die sozialen Medien im Arbeitskontext erfahren. |  |  |  |  |  |
| 10. Ich habe online sexuelle Belästigung im Arbeitskontext erfahren. |  |  |  |  |  |

**Häufigkeit von sexuellen Belästigungen und verantwortliche Personen:**

| Statement | nie | einmalig | 2-5x | >5x | Chef, Ausbilder/In | Kollege/In | Patient/In oder Angehörige | andere |
| --- | --- | --- | --- | --- | --- | --- | --- | --- |
| 1. Hatten Sie jemals das Gefühl, dass Ihr Job/Studium davon abhängt, dass Sie sexuelles Verhalten zeigen, obwohl Sie das ablehnen? |  |  |  |  |  |  |  |  |
| 2. Haben Sie jemals das Gefühl gehabt, dass Ihnen jemand körperlich unnötig nahe gekommen ist und das aus sexueller Motivation heraus? |  |  |  |  |  |  |  |  |
| 3. Haben Sie jemals Kommentare zu Ihrem Auftreten gehört, die offensichtlich einen sexuellen Bezug hatten? |  |  |  |  |  |  |  |  |
| 4. Haben Sie jemals aggressiv sexuelle Anrufe im beruflichen Kontext erhalten? |  |  |  |  |  |  |  |  |
| 5. Haben Sie jemals offensive Textnachrichten am Arbeitsplatz erhalten? |  |  |  |  |  |  |  |  |
| 6. Haben Sie jemals unerwünschte, sexuell offensive Emails am Arbeitsplatz erhalten? |  |  |  |  |  |  |  |  |
| 7. Haben Sie jemals offensive Bemerkungen über Ihre sexuelle Orientierung gesagt bekommen? |  |  |  |  |  |  |  |  |
| 8. Sollten Sie jemals unnötige und/oder wiederholte Untersuchungen tätigen, die einen Intim- bzw. Genitalbereich betrafen? |  |  |  |  |  |  |  |  |
| 9. Hat Sie jemals jemand bedrängt oder gezwungen, unerwünschten und sexuellen körperlichen Kontakt zu ihm/ihr aufzunehmen? z.B. Berühren, Küssen |  |  |  |  |  |  |  |  |
| 10. Sind Sie jemals zu sexuellem Verkehr gezwungen worden? z.B. versuchter oder durchgeführter oraler, analer oder vaginaler Verkehr |  |  |  |  |  |  |  |  |

Welches Geschlecht haben Sie bzw. welchem würden Sie sich selbst zuordnen

0 weiblich

0 männlich

0 divers (LGBTQ)

0 keines davon

Wir wüssten gerne das Geschlecht der verantwortlichen Person/en, die Sie sexuell belästigt haben?

0 männlich

0 weiblich

0 männlich und weiblich

0 keines davon

Gibt es Ihrerseits Kommentare, die uns helfen könnten, die sexuellen Belästigungen an Ihrem Arbeitsplatz nachvollziehen zu können?

Welche Ebene an Ihrem Arbeitsplatz wäre geeignet, Strategien und Maßnahmen zur Verfügung zu stellen, um weibliche Studierende und Ärztinnen sowie Schwestern vor sexuellen Übergriffen besser zu schützen?

*Kreuzen Sie alle für Sie geeigneten an*

0 keine Instanz

0 der direkte Arbeitsplatz

0 die Universität

0 das Ausbildungs- und Trainingsinstitut

0 die nationale Regierung

0 internationale Institutionen, wie z.B. die WHO

Würden bestimmte Maßnahmen als Abschreckung vor sexueller Belästigung funktionieren?

0 ja

0 nein

0 vielleicht

Welche:

Haben Sie nochmal vielen Dank für Ihre Offenheit und Unterstützung!

Bei Bedarf können Sie sich gerne an unsere Ansprechpartnerin wenden:
